# Physical Stress Induced Reduction of Proliferating Cells and Differentiated Neuroblasts Is Ameliorated by Fermented *Laminaria japonica* Extract Treatment

**DOI:** 10.3390/md18120587

**Published:** 2020-11-24

**Authors:** Hyo Young Jung, Woosuk Kim, Hyun Jung Kwon, Dae Young Yoo, Sung Min Nam, Kyu Ri Hahn, Sun Shin Yi, Jung Hoon Choi, Dae Won Kim, Yeo Sung Yoon, In Koo Hwang

**Affiliations:** 1 Department of Anatomy and Cell Biology, College of Veterinary Medicine, and Research Institute for Veterinary Science, Seoul National University, Seoul 08826, Korea; hyoyoung@snu.ac.kr (H.Y.J.); tank3430@hallym.ac.kr (W.K.); hkinging@snu.ac.kr (K.R.H.); ysyoon@snu.ac.kr (Y.S.Y.); 2 Department of Biomedical Sciences, and Research Institute for Bioscience and Biotechnology, Hallym University, Chuncheon 24252, Korea; 3 Department of Biochemistry and Molecular Biology, Research Institute of Oral Sciences, College of Dentistry, Gangneung-Wonju National University, Gangneung 25457, Korea; donuts25@gwnu.ac.kr (H.J.K.); kimdw@gwnu.ac.kr (D.W.K.); 4 Department of Anatomy, College of Medicine, Soonchunhyang University, Cheonan 31151, Korea; dyyoo@sch.ac.kr; 5 Department of Anatomy, School of Medicine and Institute for Environmental Science, Wonkwang University, Iksan 54538, Korea; namvet1@wku.ac.kr; 6 Department of Biomedical Laboratory Science, College of Medical Sciences, Soonchunhyang University, Asan 31538, Korea; admiral96@sch.ac.kr; 7 Department of Anatomy, College of Veterinary Medicine and Institute of Veterinary Science, Kangwon National University, Chuncheon 24341, Korea; jhchoi@kangwon.ac.kr

**Keywords:** stress, corticosterone, fermented *Laminaria japonica* extract, dentate gyrus, neurogenesis

## Abstract

*Laminaria japonica* is widely cultivated in East Asia, including South Korea. Fucoidan, a main component of *L. japonica*, protects neurons from neurological disorders such as ischemia and traumatic brain injury. In the present study, we examined the effects of extract from fermented *L. japonica* on the reduction of proliferating cells and neuroblasts in mice that were physically (with electric food shock) or psychologically (with visual, auditory and olfactory sensation) stressed with the help of a communication box. Vehicle (distilled water) or fermented *L. japonica* extract (50 mg/kg) were orally administered to the mice once a day for 21 days. On the 19th day of the treatment, physical and psychological stress was induced by foot shock using a communication box and thereafter for three days. Plasma corticosterone levels were significantly increased after exposure to physical stress and decreased Ki67 positive proliferating cells and doublecortin immunoreactive neuroblasts. In addition, western blot analysis demonstrated that physical stress as well as psychological stress decreased the expression levels of brain-derived neurotrophic factor (BDNF) and the number of phosphorylated cAMP response element binding protein (pCREB) positive nuclei in the dentate gyrus. Fermentation of *L. japonica* extract significantly increased the contents of reduced sugar and phenolic compounds. Supplementation with fermented *L. japonica* extract significantly ameliorated the increases of plasma corticosterone revels and decline in the proliferating cells, neuroblasts, and expression of BDNF and pCREB in the physically stressed mice. These results indicate that fermented *L. japonica* extract has positive effects in ameliorating the physical stress induced reduction in neurogenesis by modulating BDNF and pCREB expression in the dentate gyrus.

## 1. Introduction

The hippocampus in brain is related to limbic system and is closely related to learning and memory formation as well as emotions [1]. It is the most vulnerable region of the brain to ischemic damage and amyloid-β deposition [2,3]. In addition, the hippocampus is one of the most active regions in terms of the generation of new neurons throughout a person’s lifetime [4]. Stem cells in adult brain are located in the unique brain region such as subgranular zone of the dentate gyrus and are able to proliferate and differentiate into neuroblasts [4]. Newly generated mature neurons in granule cell layer contribute to hippocampus-dependent learning and memory processes [5,6,7,8]. Several lines of evidence have demonstrated that promotion of neurogenesis improves memory performance [9], while memory impairments are caused by impairments of hippocampal neurogenesis [10,11].

Stress reduces hippocampal neurogenesis in adult brains of mice and rats [12]. The cognitive performance was closely related to circulating glucocorticoid concentration in blood [13,14]. In non-human primates, stress induced by social isolation increases plasma cortisol levels and decreased cell proliferation and neuroblasts in the hippocampus [15]. In human brain, the functional connectivity is enhanced in the parahippocampal cortex with middle temporal gyrus after stress, indicating the stress affects the hippocampal memory formation in human [16]. Previously, our colleagues demonstrated that adrenalectomy significantly decreases plasma corticosterone levels, while removal of adrenal gland increases proliferating cells and neuroblasts in the dentate gyrus 5 days after surgery [17]. Increases in glucocorticoid concentration due to chronic stress exacerbates the degeneration of neurons induced by acute alcohol binging [18], the onset and progression of Alzheimer’s disease [19], and Parkinson’s disease [20]. Psychological stress does not reduce the number of neuroblasts/immature neurons in the hippocampus of ICR mice although high fat diet-fed mice presented significantly decreased neuroblasts [21]. In contrast, prenatal exposure to psychological stress significantly decreases the proliferating cells in the dorsal hippocampus [22].

*Laminaria japonica* Aresch., a brown alga, is cultivated in East Asia, including South Korea, Japan, and China. It has been consumed as a marine vegetable and has various pharmacological effects especially pertaining to neurological disorders [23,24,25] and metabolic diseases [26]. *L. japonica* contains lipophilic components such as fucoxanthin and fucosterol as well as hydrophilic fibers including alginate and fucoidan. Fucoidan, a functional component of *L. japonica*, has neuroprotective effects against ischemia, traumatic brain injury, and Parkinson’s disease [27,28,29,30]. Recently, marine seaweeds have been indicated as a new generation source for natural medicines for the treatment of neurological disorders [31,32,33,34]. Fermentation tools has great attention to enhance biological functions and to increase the yield the specific substances because fermentation with microorganism splits the complex compounds into simple ones [35,36]. Fermented *L. japonica* extract (fLJE) shows more potent antioxidant and anti-inflammatory activities compared to that in the general LJE [37,38]. Recent studies demonstrated that fermented *L. japonica* showed neuroprotective effects against scopolamine- and ethanol-induced amnesia in mice [39] as well as trimethyltin-induced memory deficits in rats [40]. However, there are currently no studies that detail the effects of fermented *L. japonica* on proliferating cells and neuroblasts in physically or psychologically stressed mice.

Therefore, in the present study, we investigated the effects of fLJE on Ki67-positive proliferating cells and doublecortin (DCX)-immunoreactive neuroblasts in the dentate gyri of mice exposed to physical or psychological stress.

## 2. Results

### 2.1. Changes of Sugar and Total Phenolic Compound Contents

Total and reducing sugar contents were significantly increased after fermentation. Especially reducing sugar contents were dramatically increased by 43.2-folds after fermentation. In addition, total phenolic compounds were significantly increased by 1.66-folds after fermentation although the statistical significance was not detected between groups (Table 1).

### 2.2. Effect of fLJE on Plasma Corticosterone Levels in Physically and Psychologically Stressed Mice

**Figure 1:**
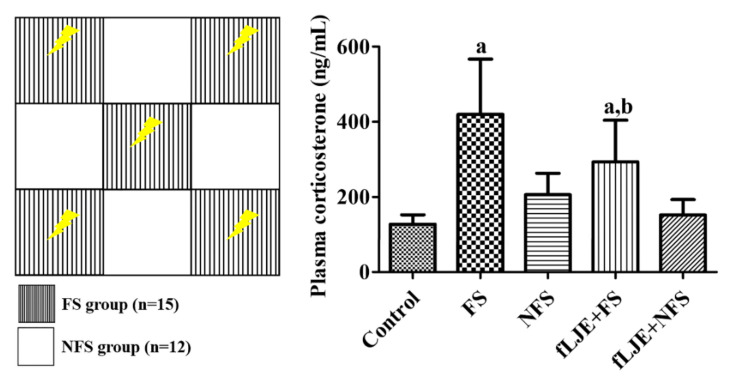
Depicted drawing of the communication box used in the present study. The electric shock is induced in FS group compartment, while in NFS group compartment there are no electric shock. However, animals feel visual, auditory and olfactory sensation from FS group. Plasma corticosterone levels in the control, foot-shock stress (FS) group, non-foot-shock stress (NFS) group, FS with fermented *Laminaria japonica* (fLJE) treatment (fLJE + FS) group, and NFS group with fLJE treatment (fLJE + NFS) (*n* = 12 or 15 per group; ^a^ *p* < 0.05, significantly different from the control group; ^b^ *p* < 0.05, significantly different between the FS and fLJE + FS groups). Bars indicate the standard deviation.

Plasma corticosterone level was 127.02 ng/mL in the control group. In the physically stressed group induced by foot-shock stress (FS group), plasma corticosterone levels dramatically increased by 3.31-fold compared to the control group, while in the psychologically stressed group induced by in non-food-shock stress (NFS group), plasma corticosterone levels did not show any significant changes compared to the control group. Statistical significance was observed only between the control and FS groups. In the fLJE-treated FS group (fLJE + FS group), plasma corticosterone levels decreased significantly down to 69.81% of the FS group. In the fLJE-treated NFS group (fLJE + NFS group), plasma corticosterone levels did not show any significant changes compared to those of the NFS group (Figure 1).

### 2.3. Effect of fLJE on Cell Proliferation in Physically and Psychologically Stressed Mice

**Figure 2:**
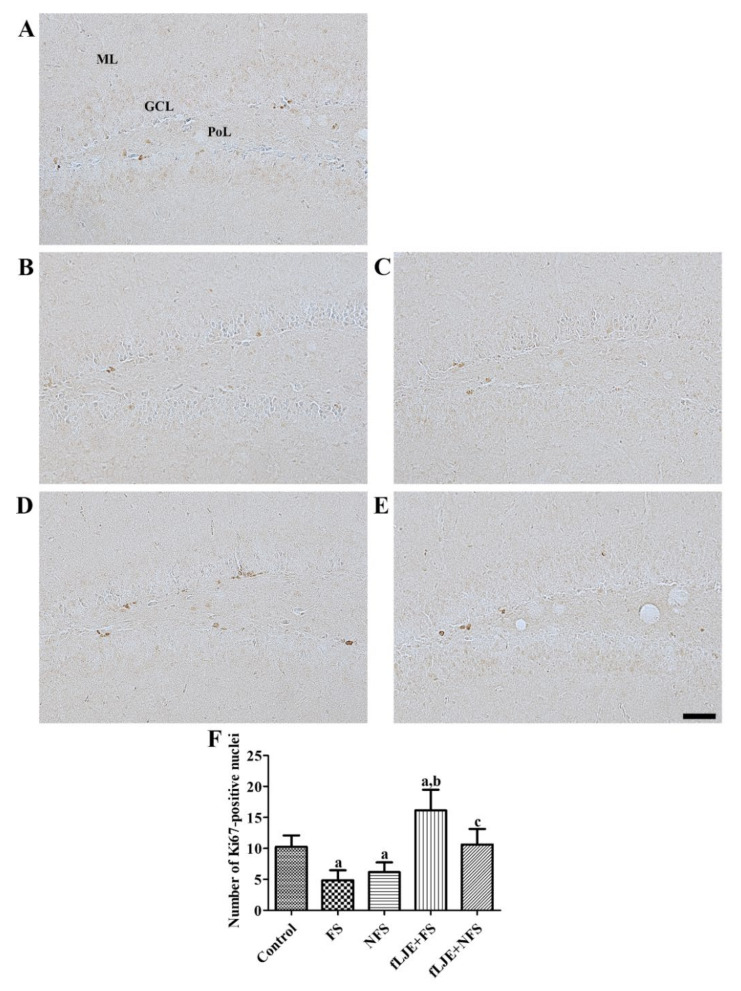
Immunohistochemistry for Ki67 in the dentate gyrus of the control (**A**), foot-shock stress (FS) group (**B**), non-food-shock stress (NFS) group (**C**), FS with fermented *Laminaria japonica* (fLJE) treatment (fLJE + FS) group (**D**), and NFS group with fLJE treatment (fLJE + NFS) (**E**), GCL, granule cell layer; ML, molecular layer; PoL, polymorphic layer. Scale bar = 50 μm. (**F**) The mean number of Ki67-positive cells per section in all the groups (*n* = 6 or 8 per group; ^a^ *p* < 0.05, significantly different from the control group; ^b^ *p* < 0.05, significantly different between the FS and fLJE + FS groups; ^c^ *p* < 0.05, significantly different between the NFS and fLJE + NFS groups). Bars indicate the standard deviation.

In the control group, proliferating cells expressing Ki67 were detected mainly in the subgranular zone of the dentate gyrus (Figure 2A). In this group, the mean number of Ki67-positive nuclei in the dentate gyrus was 10.24 per section (Figure 2F). In the FS and NFS groups, significantly fewer Ki67-positive nuclei were found in the subgranular zone of the dentate gyrus compared to the control group and the mean number of Ki67-positive nuclei was 4.85 and 6.15 per section, respectively (Figure 2B,C,F). In the fLJE-FS and fLJE-NFS groups, Ki67-positive nuclei were abundantly observed in the subgranular zone of the dentate gyrus and the number was significantly higher (332.73% and 172.63%, respectively) in these groups than in the FS and NFS groups (Figure 2D–F).

### 2.4. Effect of fLJE on Neuroblast Differentiation in Physically and Psychologically Stressed Mice

**Figure 3:**
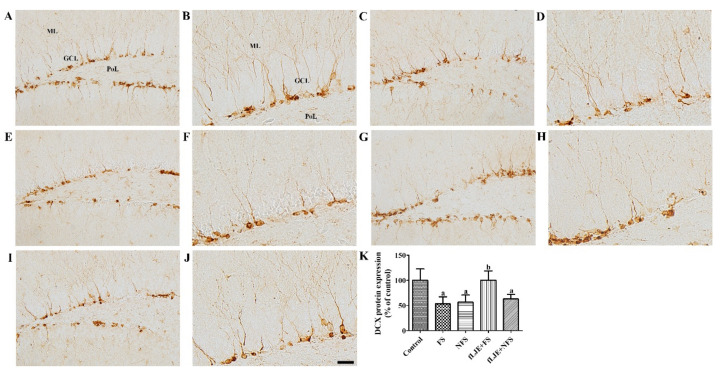
Immunohistochemistry for doublecortin (DCX) in the dentate gyrus of the control (**A**) and (**B**), foot-shock stress (FS) group (**C**) and (**D**), non-food-shock stress (NFS) group (E and F), FS with fermented *Laminaria japonica* (fLJE) treatment (fLJE + FS) group (**G**) and (**H**), and NFS group with fLJE treatment (fLJE + NFS, I and J). GCL, granule cell layer; ML, molecular layer; PoL, polymorphic layer. Scale bar = 50 μm (**A**,**C**,**E**,**G**,**I**) or 25 μm (**B**,**D**,**F**,**H**,**J**). (**K**): The relative optical densities (RODs) expressed as a percentage of the value representing the DCX immunoreactivity in the dentate gyrus of the control group are shown (*n* = 6 or 8 per group; ^a^ *p* < 0.05, significantly different from the control group; ^b^ *p* < 0.05, significantly different between FS and FS-fLJE groups). Bars indicate the standard deviation.

In the control group, differentiated neuroblasts expressing DCX were observed in the dentate gyrus. Their cytoplasm was located in the subgranular zone of the dentate gyrus and their dendrites extended into the molecular layer of the dentate gyrus (Figure 3A,B). In the FS and NFS groups, DCX-immunoreactive neuroblasts were less abundantly detected in the dentate gyrus compared to the control group (Figure 3C–F). DCX immunoreactivity in these groups was significantly decreased by 53.35% and 56.57% of the control group, respectively (Figure 3K). In the fLJE + FS group, DCX-immunoreactive neuroblasts were abundantly detected in the dentate gyrus (Figure 3G,H). DCX immunoreactivity in this group was significantly increased compared to the FS group and was similar to that of the control group (Figure 3K). In the fLJE + NFS group, DCX immunoreactivity was similar to that in the NFS group (Figure 3I–K).

### 2.5. Effect of fLJE on Phosphorylated Camp Response Element Binding Protein (Pcreb) Expression in Physically and Psychologically Stressed Mice

**Figure 4:**
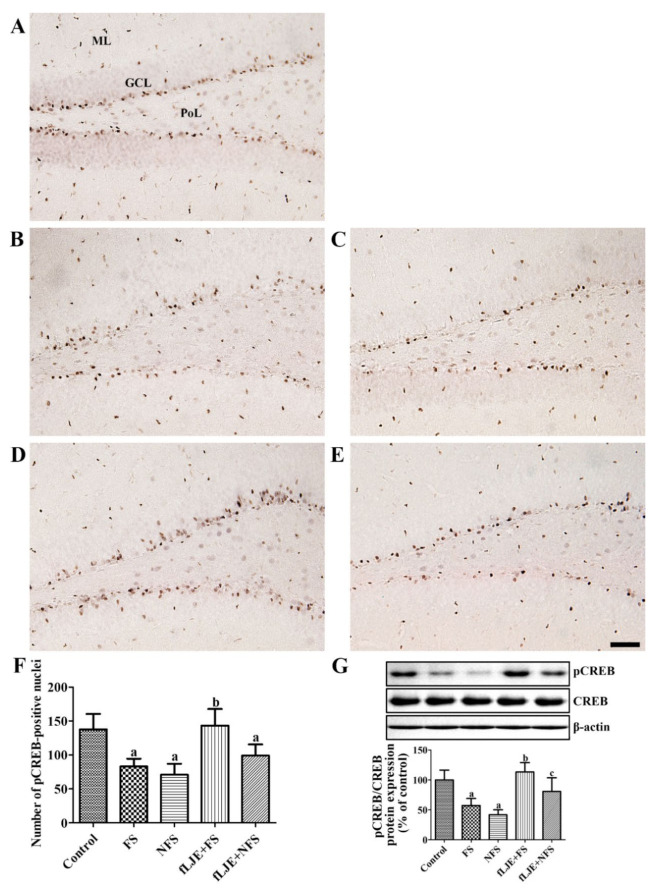
Immunohistochemistry for phosphorylated cAMP response element binding protein (pCREB) in the dentate gyrus of the control (**A**), foot-shock stress (FS) group (**B**), non-food-shock stress (NFS) group (**C**), FS with fermented *Laminaria japonica* (fLJE) treatment (fLJE + FS) group (**D**), and NFS group with fLJE treatment (fLJE + NFS, (**E**). GCL, granule cell layer; ML, molecular layer; PoL, polymorphic layer. Scale bar = 50 μm. (**F**): The mean number of pCREB-positive cells per section in all groups is shown. (**G**): Western blot analysis, expressed as a percentage of the value of the phosphorylated cAMP response element binding protein (pCREB) and CREB immunoblot bands of the control group, respectively. Data were represented to the percentage of pCREB/CREB ratio in each lane (*n* = 5 or 7 per group; ^a^ *p* < 0.05, significantly different from the control group; ^b^ *p* < 0.05, significantly different between FS and FS-fLJE groups; ^c^ *p* < 0.05, significantly different between the NFS and fLJE + NFS groups). Bars indicate the standard deviation.

In the control group, pCREB immunoreactivity was observed in the dentate gyrus, and was especially abundant in the subgranular zone of the dentate gyrus (Figure 4A). In this group, the mean number of pCREB-positive nuclei was 137.74 in the subgranular zone of the dentate gyrus per section (Figure 4F). In the FS and NFS groups, pCREB-positive nuclei were less abundant in the dentate gyrus compared to the control group (Figure 4B,C). In these groups, the number of pCREB-positive nuclei in the subgranular zone of the dentate gyrus was 60.31% and 51.44% of the control group, respectively (Figure 4F). In the fLJE + FS group, pCREB-positive nuclei were abundant in the subgranular zone of the dentate gyrus and the number was significantly increased compared to the FS group (Figure 4D,F). In the fLJE + NFS group, more pCREB-positive nuclei were found in the subgranular zone of the dentate gyrus compared to the NFS group although statistically significant difference were not detected (Figure 4E,F). Western blot analysis showed similar levels of CREB protein in all groups, while pCREB/CREB protein levels in the FS and NFS groups were significantly decreased down to 57.4% and 41.6% of control group, respectively. However, pCREB/CREB protein levels were significantly increased in the fLJE + FS group by 1.97-fold of FS group and similar level to control group. In the fLJE + NFS group, pCREB/CREB protein levels were also significantly increased by 1.94-fold of NFS group (Figure 4G).

### 2.6. Effect of fLJE on BDNF Expression in Physically and Psychologically Stressed Mice

**Figure 5:**
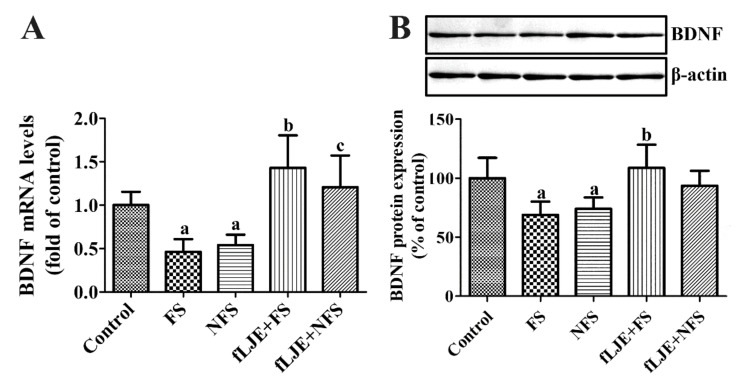
Quantitative real-time polymerase chain reaction (**A**) and western blot analysis (**B**) in the control, foot-shock stress (FS) group, non-food-shock stress (NFS) group, FS with fermented *Laminaria japonica* (fLJE) treatment (fLJE + FS) group, and NFS group with fLJE treatment (fLJE + NFS), expressed as a relative amount of brain-derived neurotrophic factor (BDNF) mRNA and percentage of the value of the BDNF immunoblot band of the control group. Data from quantitative real-time polymerase chain reaction were analyzed using the method of 2^-^^ΔΔ^Ct and presented relative to the control group and western blot data were normalized to β-actin levels in each lane (*n* = 5 or 7 per group; ^a^ *p* < 0.05, significantly different from the control group; ^b^ *p* < 0.05, significantly different between FS and fLJE + FS groups; ^c^ *p* < 0.05, significantly different between the NFS and fLJE + NFS groups). Bars indicate the standard deviation.

In the FS group, BDNF mRNA and protein levels in the hippocampal dentate gyrus were significantly decreased down to 46.3% and 68.93% compared to the control group, respectively. In the NFS group, BDNF mRNA and protein levels showed similar reduction (down to 54.1% and 74.05% of control group) compared to the control group, respectively. In the fLJE + FS group, BDNF mRNA and protein levels were increased by 3.09 and 1.57-folds of FS group, respectively, and were slightly higher than in the control group. In the fLJE + NFS group, BDNF mRNA level was significantly increased compared to the NFS group, but no changes of BDNF levels were detected in the hippocampal homogenates between NFS and fLJE + NFS group (Figure 5).

## 3. Discussion

Transient mild to moderate stress enhances the compensatory adaptive ability to overcome stressful events, while long-lasting stress causes various problems including mental disorders [41,42,43]. In the present study, we used a communication box to induce physical and psychological stress in mice and examined the effects of fLJE on plasma corticosterone levels in these mice. Physical stress elevated plasma corticosterone levels by 3.31-fold compared to those in the control group, while plasma corticosterone levels did not show significant changes in psychologically stressed mice. This result is partially consistent with previous studies indicating that serum corticosterone levels are significantly increased in both physically and psychologically stressed groups compared to their control groups [44,45]. This discrepancy may be associated with the susceptibility of males and females to chronic stress because neurochemical profiling shows that females are more vulnerable to stress than males [46,47,48,49]. Other groups demonstrated that psychological stress using a communication box increased acutely blood corticosterone levels but did not change after 15 or 30 days-stress exposure [45,50]. Repeated injection stress for 6 weeks increased corticosterone reactivity, while the social isolation decreased the corticosterone reactivity [51]. We assumed that corticosterone is more reactive to physical stress than psychological stress and we observed the significant reduction of corticosterone levels in physical, not psychological stress.

Enhancement of hippocampal neurogenesis decreases corticosterone-induced anxiety and depression-related behaviors in mice [52]. In contrast, ablation of hippocampal neurogenesis in mice shows reduced anxiety-like behavior during the dark cycle and exacerbates the decreases in corticosterone levels after restraint stress [53]. In the present study, we observed the effects of fLJE on cell proliferation and neuroblast differentiation in physically and psychologically stressed mice. Physical and psychological stress significantly decreased the number of proliferating cells and neuroblasts in the dentate gyrus compared to the control group. Predator stress significantly reduced the number of bromodeoxyuridine and DCX double positive cells in the dentate gyrus compared to the control group [54]. In addition, early life stress significantly decreased the number of proliferating cells and neuroblasts in the dentate gyrus [55]. There is a contradictory report indicating that DCX-immunoreactive neuroblasts are similarly observed in control and psychologically stressed mice 7 days after the last stress session while high fat diet-fed ICR mice showed significant decreases in DCX-immunoreactive neuroblasts 7 days after the last stress session compared to high diet-fed control mice [21]. This discrepancy may be associated with the duration and intensity of the electric shocks, and the euthanization time after the last stress session. In the present study, the administration of fLJE significantly increased the number of proliferating cells and neuroblasts in physically stressed mice, but not in the psychologically stressed mice. Several studies have demonstrated that red, green, and brown algae enhance neurogenesis [56,57,58]. In addition, fucoidan derived from *Fucus vesiculosus* and *Undaria pinnatifida* promoted neurite outgrowth in PC12 cells [59].

In the present study, we examined the expression of mature BDNF in the hippocampus to find the possible mechanisms associated with the effects of fLJE on cell proliferation and neuroblast differentiation because stress-induced glucocorticoid regulates BDNF expression [60,61] and BDNF is related to alterations due to psychological stress and synaptic plasticity [62,63,64]. Genetic deletion of BDNF impairs synaptic plasticity based on long-term potentiation [65], while BDNF supplementation or overexpression significantly ameliorates the impairment in synaptic function [66,67]. In addition, several lines of evidence demonstrate that some doublecortin cells expressed the tropomyosin receptor kinase B [68,69,70]. In the present study, we sacrificed the animals immediately after the last stress session because BDNF levels dynamically change after stress [71]. Physical and psychological stress significantly decreased BDNF levels in the hippocampus in our study. This result is consistent with previous studies that showed acute immobilization and restraint stress reduced BDNF mRNA levels in the dentate gyrus [60,71,72,73]. In addition, fear conditioning and social isolation decreased mRNA expression in the hippocampus [74,75]. Moreover, Li et al. showed that psychological stress significantly decreased BDNF mRNA levels immediately after stress and that BDNF mRNA levels returned to baseline 2 h after stress [71]. Administration of fLJE to physically stressed mice significantly increased BDNF expression compared to the FS group. However, we could not observe the changes of BDNF levels in the hippocampus of psychologically stressed group after fLJE treatment. In a model of memory deficit induced by trimethyltin treatment, administration of 50-200 mg/kg fLJE recovered the reduction in BDNF expression in the hippocampus [40]. Fucoidan also ameliorated the reduction in BDNF expression in the hippocampus [73].

In the present study, we also observed pCREB immunoreactivity in the dentate gyrus, because CREB is believed to be one of the components of the BDNF downstream pathway [76,77]. Moreover, the phosphorylation of CREB at Ser133 facilitates neuronal regeneration and repair [78,79,80]. In the present study, we observed a significant reduction in pCREB-positive nuclei after physical and psychological stress in mice. Consistent with the changes in BDNF expression in the hippocampus, the number of pCREB-positive nuclei in the fLJE-FS group was significantly increased compared to the FS group, while pCREB was moderately (not significantly) increased in fLJE-NFS group compared to the NFS group.

Enhanced neurogenesis by environmental enrichment, electroacupuncture, and fluoxetine treatment significantly improved neurogenesis and the expression of BDNF and pCREB in the hippocampus [81,82]. In contrast, intracerebroventricular infusion of K252a, a Trk antagonist, blocked the enhanced neurogenesis in the dentate gyrus [81,82] and enhanced expression of pCREB [82]. Administration of fLJE mitigated the reduction in pCREB immunoreactive cells in the hippocampus after trimethytin treatment [40]. However, Park et al. only observed BDNF and pCREB immunoreactivity in the hippocampal CA1 and CA3 regions, but not in the dentate gyrus [40]. In the present study, we observed the effects of fLJE on cell proliferation and neuroblast differentiation in the hippocampus of physically stressed mice, not psychologically stressed mice, in relation to the expression of BDNF and pCREB.

Recently, many studies have been conducted to elucidate the active ingredients in LJE. The LJE extract contains alginates, fucoidan, laminarin, and fucoxanthin with anti-viral, anti-tumor, anti-hyperglycemic, and anti-coagulation activities [26,83,84]. In the present study, we observed the dramatic increases of reducing sugar contents after fermentation of LJE and the total phenolic compounds were also significantly increased. The fermentation of LJE by *A. oryzae* may cause disruption of seaweed cell wall and increased phenolic compounds molecules because *A. oryzae* contains high amounts of catalytic enzymes [85]. This fermentation may increase the phenolic compounds to activate the cell proliferation and neuroblast differentiation in the dentate gyrus. Brain has blood-brain barrier, which is a unique structure to limit the delivery of active compounds into brain. Many studies have been attempted to find the possible molecules to cross the blood-brain barrier in LJE and fLJE. Fucoidan, an abundant compound derived from marine brown algae, has anti-depressant effects and ameliorated the reduction of BDNF-dependent synaptic plasticity in the hippocampus [86]. In addition, fucoidan from *Ecklonia cava* showed improvement from learning and memory impairment induced by trimethyltin treatment [87]. In the memory deficit model induced by D-galactose treatment, secondary metabolites of *Galactomyces geotrichum* from *Laminaria japonica* shows positive effects on cognitive function [88]. However, the study has limitation because we did not observe the possible polyphenolic compounds or fucoidan to cross the blood-brain barrier and to ameliorate the stress-induced reduction of hippocampal neurogenesis and BDNF-pCREB signaling.

In conclusion, physical stress, not psychological stress, significantly increases plasma corticosterone levels. However, physical, not psychological, stress decreases the number of proliferating cells, neuroblasts, pCREB-positive nuclei, and BDNF expression in the dentate gyrus. Administration of fLJE significantly ameliorates the changes occurring in physically, not psychologically, stressed mice. These results suggest that fLJE can be used as a functional food to improve the physical, not psychological, stress induced reduction in hippocampal neurogenesis by increasing BDNF and pCREB levels when consumed equivalent dosage (486 mg) converted in human based on the equation described by Nair and Jacob [89].

## 4. Materials and Methods

### 4.1. Preparation of fLJE

*L. japonica* was obtained from the local market in Jeju and we freeze-dried it to ferment with *Aspergillus oryzae* at 35 ± 1 °C for 72 h. Thereafter, the fermented *L. japonica* was extracted with water-extraction method. Ethanol is added to extract solution and centrifuged to separate the ethanol-soluble fraction, which is lyophilized and store at −80 °C until utilization.

### 4.2. Total and Reducing Sugar Contents

Total sugar in LJE were measured spectrophotometrically before and after fermentation by phenol-sulfuric method. In addition, the reducing sugar contents were analyzed by enzymatic methods using 3,5-dinitrosalicylic acid method described by Marsden et al. [90].

### 4.3. Total Phenolic Assay

Total phenols in the fLJE were measured by using Folin-Ciocalteu reagent. In brief, the mixture of 0.5 mL of fLJE and 0.1 mL of Folin-Ciocalteu reagent were made and 2.5 mL of saturated Na_2_CO_3_ (75 g/L) was added for 30 min at 25 °C. The absorbance was measured at 760 nm using spectrophotometer. The concentration of the total phenolic compounds was calculated as mg of gallic acid equivalent by using an equation obtained from gallic acid calibration curve.

### 4.4. Experimental Animals

Sixty-nine male Institute Cancer Research (ICR, CD-1) outbred mice (5 weeks of age) were obtained from Orient Bio (Seongnam, South Korea) and the mice were housed in a conventional facility at Seoul National University and experimental protocols were approved by the Institutional Animal Care and Use Committee of Seoul National University (SNU-120103-10).

### 4.5. Experimental Groups

The animals were divided into 5 groups as follows: control (*n* = 15), physical stress (foot-shock, FS) group (*n* = 15), psychological stress (non-foot-shock, NFS) group (*n* = 12), 50 mg/kg fLJE-treated FS (fLJE-FS) group (*n* = 15), 50 mg/kg fLJE-treated NFS (fLJE-NFS) group (*n* = 12). Six-week-old mice received an oral administration of 100 mg/kg fLJE once a day for 21 days because neuroblasts/immature neurons are expressed in DCX from day 1 to day 28 of birth in cells [91,92]. The dosage of fLJE was chosen because Reid et al. showed cognitive improvements at a dose of 50 mg/kg of fLJE [39].

### 4.6. Induction of Physical or Psychological Stress

On day 19 of the treatment, physical or psychological stress was induced using a communication box as described by Li et al. [93]. The communication box consisted of an open box (48 cm × 48 cm × 50 cm) with transparent acryl as shown in Figure 1. Stainless steel rods placed on the floor to administer electrical shocks at the center and in the four corners (physical stress compartment) and a plastic insulator set at the others (psychological stress compartment). Mice were randomly divided into FS and NSF group and placed in each compartment for adaptation to the apparatus 30 min prior to the induction of stress. Five (*n* = 30 in total) of the nine mice (*n* = 54 in total) located in physical stress compartment received a 0.3-mA electric current for 10 s. The electric shock was delivered randomly for an average of 30 times in 60 min using a shock generator (Jeung Do Bio & Plant Co., LTD, Seoul, Korea) between 14:00 and 15:00 for three consecutive days. The other four (NFS) mice (*n* = 24 in total) experienced the stimuli (visual, auditory and olfactory sensation) from physically stressed mice. After last electric shock, mice returned to its original position on the cage and were provided with both water and food *at libitum*. The control mice (*n* = 15) were placed in electric-free compartment with electric stimulation in the absence of the physically stressed mice. All mice were carefully handled during the experiment to minimize the stress described by Gouveia and Hurst [94].

### 4.7. Blood Sampling and Measurements of Plasma Corticosterone Levels

Following the physical and psychological stress episodes, animals (*n* = 12 or 15 in each group) were deeply anesthetized with a mixture of alfaxalone (Alfaxan, 75 mg/kg; Careside, Seongnam, Korea) and xylazine (10 mg/kg; Bayer Korea, Seoul, Korea), and blood samples were obtained from cardiac puncture in each animal. The plasma was separated from the blood and the plasma corticosterone levels were measure as described in a previous study [95].

### 4.8. Tissue Processing for Histology and Immunohistochemical Staining

After blood sampling, the animals were perfused transcardially and brain sample was dissected as described previously [96,97]. Thirty micrometer brain sections were cut between 1.82 and 2.30 mm caudal to the bregma, as defined by a mouse atlas [98] and immunohistochemical staining for Ki67, DCX, and phosphorylated cAMP response element binding protein at Ser133 (pCREB) was conducted as described in a previous study [96]. Briefly, the tissue sections located at a distance of 120 μm from each other were selected and incubated with rabbit anti-Ki67 antibody (1:1000; Abcam, Cambridge, UK), rabbit anti-DCX (1:5000; Abcam), or rabbit anti-pCREB (1:400; Cell Signaling Technology, Inc., Beverly, MA, USA) at 25 °C overnight. The sections were sequentially treated with biotinylated goat anti-rabbit IgG and a streptavidin-peroxidase complex (1:200; Vector, Burlingame, CA, USA) for 2 h at 25 °C. The sections were visualized by reacting 3,3’-diaminobenzidine tetrachloride (Sigma) in 0.1 M Tris-HCl buffer (pH 7.2). The specificity of antibodies used in the present study was established by substitution of goat anti-rabbit IgG to isotype control IgG (Abcam) [99]. Cancer tissues from xenograft tissue were also used for Ki67 as a positive control. Immunoreactive structure disappeared completely in dentate gyrus and Ki67 immunoreactive cells are abundantly detected in cancer tissues (Figure S1).

### 4.9. Western Blot and Quantitative Real-Time Polymerase Chain Reaction (PCR)

Following the last physical and psychological stress episode, the animals (*n* = 6–7 in each group) were immediately sacrificed using with a mixture of alfaxalone and xylazine. After subsequent decapitation, the 500-µm-thick hippocampal sections were used for western blot and real-time PCR study as described in a previous study [87]. Briefly, the protein-transferred membrane was sequentially incubated with rabbit anti-BDNF (diluted 1:5000, Abcam), CREB (1:1000; Cell Signaling Technology, Inc., Beverly, MA, USA), or pCREB (1:1000; Cell Signaling Technology, Inc.), peroxidase-conjugated goat anti-rabbit IgG (1:5000, SantaCruz Biotechnology, Santa Cruz, CA, USA), and an ECL chemiluminescent kit (Pierce; Thermo Fisher Scientific, Inc., Waltham, MA, USA). For quantitative real-time PCR analyses, the hippocampal tissues were processed as described by Cao et al. [100] and primers of BNDF were used as described in the previous study [97].

### 4.10. Data Analysis

DCX immunoreactivity was quantitatively analyzed using ImageJ software v. 1.50 (National Institutes of Health, Bethesda, MD, USA) as described in previous studies [15,86,87]. Digital image of DCX immunoreactive structures was converted into gray, which has a resolution with 0-255 and the gray intensity and pixel number was measured. Intensity of DCX immunoreactivity was evaluated by determining the relative optical density (ROD), which was obtained after transformation of the mean gray level using the following formula: ROD = log_10_ (256/mean gray level).

The number of Ki67- and pCREB-immunoreactive nuclei was counted with OPTIMAS software (version 6.5; CyberMetrics^®^ Corporation, Phoenix, AZ, USA) as described in a previous study [96].

### 4.11. Statistical Analysis

Data was analyzed statistically by one-way analysis of variance followed by Bonferroni’s post-hoc test using GraphPad Prism 5.01 software (GraphPad Software, Inc., La Jolla, CA, USA) and the results were considered to be statistically significant if *p* < 0.05.

## Figures and Tables

**Table 1:** Contents of total and reducing sugars as well as total phenolic compounds are analyzed by spectrophotometric methods before and after fermentation of LJE.

Sample	Total Sugars (mg/mL)	Reducing Sugars (mg/mL)	Total Phenolic Compounds (Μg/mL)
Before (LJE)	3.51 ± 0.29	4.85 ± 2.69	2.48 ± 0.32
After (fLJE)	6.02 ± 0.69 ^a^	209.51 ± 16.52 ^a^	4.11 ± 0.25 ^a^

Values are mean ± standard deviation (*n* = 5; ^a^ *p* < 0.05, significantly different from the LJE group).

## Supplementary Materials

The following are available online at https://www.mdpi.com/1660-3397/18/12/587/s1, Figure S1: Control staining for antibodies used in this study.
