# Supplementary material for: Physical Stress Induced Reduction of Proliferating Cells and Differentiated Neuroblasts Is Ameliorated by Fermented Laminaria japonica Extract Treatment
Source: Mar Drugs. 2020 Nov 24;18(12):587. doi: 10.3390/md18120587 (PMC7760277; doi:10.3390/md18120587)
Supplement: Supplementary file 1 [file marinedrugs-18-00587-s001.pdf]

Physical Stress Induced Reduction of Proliferating Cells and Differentiated Neuroblasts is Ameliorated by Fermented Laminaria japonica Extract Treatment

Supplementary Material

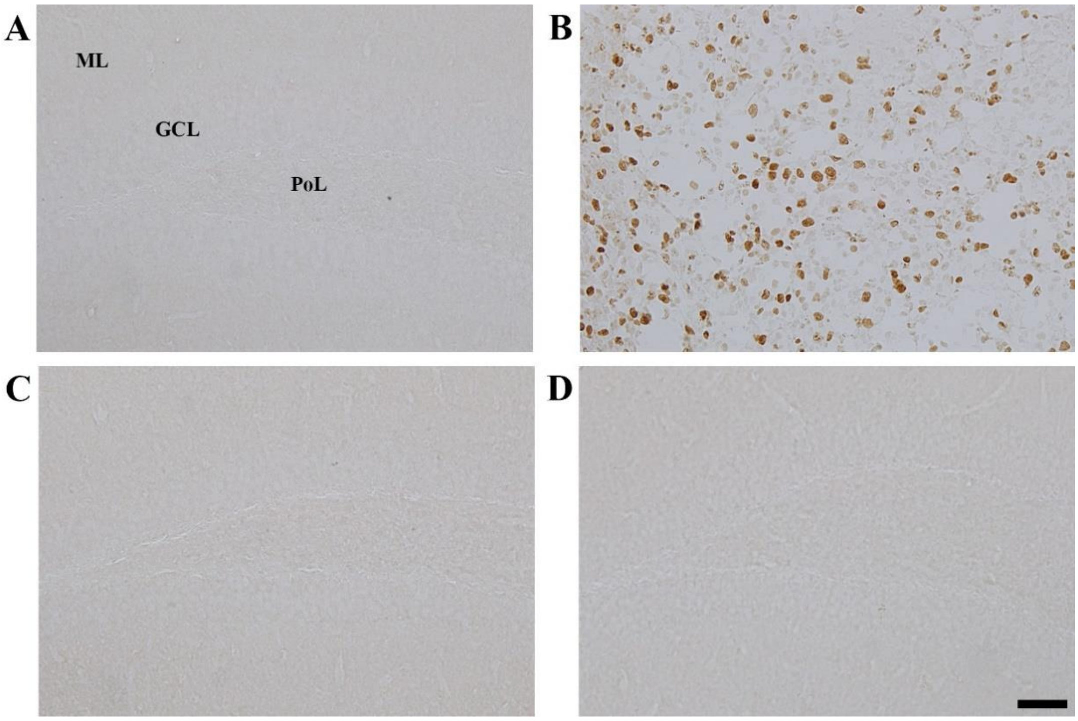

Figure S1. Negative control immunohistochemical staining for Ki67 (**A**), doublecortin (DCX, **C**), and phosphorylated cAMP response element binding protein (pCREB, **D**) with substitution of goat anti-rabbit IgG to isotype control IgG in the hippocampus of the control group. Positive staining for Ki67 (**B**) is also shown using cancer tissue. No Ki67, DCX, and pCREB immunoreactive structures are shown in the hippocampal tissue, while numerous Ki67 positive cells are detected in the cancer tissue. GCL, granule cell layer; ML, molecular layer; PoL, polymorphic layer. Scale bar = 50 μm.
